# External quality assessment (EQA) for tumor mutational burden: results of an international IQN path feasibility pilot scheme

**DOI:** 10.1007/s00428-022-03444-y

**Published:** 2022-11-10

**Authors:** Riziero Esposito Abate, Melanie H. Cheetham, Jennifer A. Fairley, Raffaella Pasquale, Alessandra Sacco, Wolstenholme Nicola, Zandra C. Deans, Simon J. Patton, Nicola Normanno

**Affiliations:** 1 Cell Biology and Biotherapy Unit, Istituto Nazionale Tumori “Fondazione G. Pascale”-IRCCS, Naples, Italy; 2 European Molecular Genetics Quality Network (EMQN), Unit 4, Enterprise House, Pencroft Way, Manchester Science Park, Manchester, M15 6SE UK; 3 GenQA, Department of Laboratory Medicine, Royal Infirmary of Edinburgh, Little France Crescent, Edinburgh, EH16 4SA UK

**Keywords:** External quality assessment, Predictive biomarkers, Tumor mutational burden, TMB testing

## Abstract

Tumor mutational burden (TMB) has recently been approved as an agnostic biomarker for immune checkpoint inhibitors. However, methods for TMB testing have not yet been standardized. The International Quality Network for Pathology (IQNPath) organized a pilot external quality assessment (EQA) scheme for TMB testing. The aim of this program was the validation of the materials and the procedures for the EQA of this complex biomarker. Five formalin-fixed paraffin-embedded (FFPE) cell lines were selected to mimic the various TMB values observed in clinical practice. The FFPE samples were tested with the FoundationOne CDx (F1CDx) assay as the reference test and three commercially available targeted sequencing panels. Following this internal validation, the five cell lines were sent to 29 laboratories selected on the basis of a previous survey. Nineteen of the 23 laboratories that submitted results (82.6%) used targeted sequencing for TMB estimation. Only two laboratories performed whole exome sequencing (WES) and two assessed TMB by clinical exome. A high variability in the reported TMB values was observed. The variability was higher for samples with the highest TMB value according to the F1CDx test. However, good reproducibility of the TMB score was shown by laboratories using the same panel. The majority of laboratories did not indicate a TMB cut-off value for clinical interpretation. In conclusion, this pilot EQA scheme suggests that it is feasible to run such an EQA program for TMB assessment. However, the results of our pilot highlight the numerous challenges for the standardization of this test.

**Supplementary Information:**

The online version contains supplementary material available at 10.1007/s00428-022-03444-y.

## Introduction

The introduction of immune checkpoint inhibitors (ICIs) has significantly improved clinical outcomes and quality of life of patients with different cancer types [1]. Several different ICIs have already been approved for clinical practice or are in an advanced phase of clinical investigation. These compounds include molecules targeting programmed cell death receptor-1 (PD-1) or its ligand (PD-L1) as well as cytotoxic T-lymphocyte antigen 4 (CTLA-4), which play a critical role in T-cell activation and tumor evasion [2].

Extensive efforts are being made to identify robust biomarkers to select immune-responsive patients. Microsatellite instability (MSI) or alterations in the mismatch repair system (dMMR) correlate with sensitivity to ICIs, whereas immunohistochemical detection of PD-L1 can enrich for immune-responsive patients at least in some tumor types [3]. In recent years, tumor mutational burden (TMB), defined as the number of somatic mutations per Megabase (mut/Mb) in the coding area of the genome, has been evaluated as a biomarker for ICIs in different tumor types, including non-small cell lung cancer (NSCLC), melanoma, and bladder cancer [4–6]. The hypothesis supporting TMB as a biomarker for ICIs is that somatic mutations may lead to the formation of tumor-specific neoantigens, which are able to trigger T-cell activation against tumor cells [7–10]. There is a huge variability in mutation load among individual tumors and within tumor types, which reflects exposures to mutagenic agents such as tobacco and UV or alterations in genes involved in the mechanisms of DNA repair and synthesis [1, 11, 12]. In agreement with this hypothesis, clinical trials showed that patients with high TMB values had a better response and outcome as compared with patients with low TMB, when treated with single agent or combination of PD-L1 and CTLA inhibitors [13–15]. More importantly, TMB was approved by the Food and Drug Administration (FDA) as an agnostic biomarker for the treatment with pembrolizumab of adult and pediatric patients with unresectable or metastatic TMB high (≥ 10 mut/Mb) solid tumors based on the results of the KEYNOTE-158 trial [16–18]. While these findings demonstrate an increasing interest in the assessment of TMB across different tumor types, TMB testing is far from being standardized. The eligible method to assess this potential biomarker is whole exome sequencing (WES), but the costs, the turnaround time, and the necessary minimum coverage make this approach largely unfeasible in a clinical practice context.

TMB quantification with targeted next generation sequencing (NGS) panels has been shown to correlate with WES-derived TMB and to associate with ICI response, making the clinical assessment of TMB practically achievable. Several different panels covering at least 1 Mb of the coding regions of the genome have been used to calculate TMB [1, 19, 20]. However, the application of NGS in clinical practice can be challenging due to the use of formalin-fixed paraffin-embedded (FFPE) blocks as diagnostic samples. In fact, formalin fixation can significantly affect the quality and the quantity of the DNA extracted and can also lead to the creation of deamination artifacts [21, 22]. In addition, the TMB value can be affected by the choice of genes covered and bioinformatics platform.

FoundationOne CDx (F1CDx) and MSK-Impact are the only two targeted sequencing panels currently approved by the US Food and Drug Administration to assess TMB [23, 24] with the F1CDx test used for TMB estimation in the KEYNOTE 158 trial [16]. Different commercial companies have recently developed several assays for TMB evaluation using several approaches in terms of gene panel composition, type of sequencing technology, and bioinformatics pipeline [1, 19, 25].

However, the optimal TMB cut-off to discriminate potential responder’s vs non-responders to ICIs may vary between different panels and among cancer types [26]. In this respect, the Friends of Cancer Research (FOCR) TMB Harmonization Project, an important public–private initiative, recently published the results of the characterization of panel-based variability in TMB estimation [27, 28].

With these points and initiatives in mind, the International Quality Network of Pathology (IQN Path), a network of quality assessment associations with an interest in cancer biomarker testing, organized a pilot external quality assessment (EQA) scheme for TMB testing with the collaboration of different academic partners (AIOM, Gen&Tiss, ESP, GenQA, EMQN, cIQc, RCPA Quality Assurance Programs). The main aim of this pilot EQA was the validation of the materials and the procedures for the EQA of this complex biomarker.

## Materials and methods

In this section, we describe all materials and methods used in the internal validation of the materials used in this EQA pilot scheme.

### FFPE cell lines and genomic DNA extraction

The study included nine cell lines provided as FFPE samples from Seracare (LGC Clinical Diagnostics, Inc.) (*NCI-H2009*; *NCI-H2126*; *NCI-H2171*; *NCI-H1437*; *NCIH23*; *NCIH322*; *SKMEL2*; *C33A*; *IGROV1*). Genomic DNA (gDNA) was isolated from two 10 µm-thick FFPE tissue sections using the GeneRead DNA FFPE kit (Qiagen) according to the manufacturer’s protocol. The gDNA quantity was assessed with the Qubit dsDNA HS assay kit (Thermo Fisher Scientific) using the Qubit 2.0 Fluorometer (Thermo Fisher Scientific). The samples were tested with three commercially available NGS targeted panels: Oncomine Tumor Mutation Load (OTML) Assay (Thermo Fisher Scientitic), TruSight Oncology 500 (TSO500) Assay (Illumina), QIAseq® Targeted DNA Panel (Qiagen), and the F1CDx test.

#### Oncomine tumor mutation load assay testing

The Oncomine Tumor Mutation Load Assay (OTML) covers a coding sequence region of 1.18 Mbp that include 409 genes with known cancer associations (Thermo Fisher Scientific). Libraries were prepared using Ion AmpliSeq™ Library Kit Plus (Thermo Fisher Scientific) starting from 10 ng of gDNA for each pool. Barcoded libraries were quantified with the Ion Library TaqMan® Quantitation Kit (Thermo Fisher Scientific). Fifty picomoles of each library were multiplexed and clonally amplified by emulsion PCR, performed and enriched on the Ion Chef™ instrument (Thermo Fisher Scientific). Finally, the template was loaded on an Ion 540™ Chip and sequenced on an Ion S5™ XL sequencer (Thermo Fisher Scientific) according to the manufacturer’s instructions. Each sequenced chip contained 4 samples.

#### TruSight oncology 500 assay

The TruSight Oncology 500 Assay (TSO500) analyzes 523 genes in a coding region of 1.3 Mb (Illumina). Samples were fragmented with a M-220 Focused-ultrasonicator (Covaris) using 80 ng of gDNA. To evaluate the peak size of the generated fragments, the samples were run on the 2100 Bioanalyzer Instrument (Agilent) using the High Sensitivity DNA Kit (Agilent). Libraries were prepared following the manufacturer’s instructions and were quantified with the Qubit dsDNA HS Assay Kit (Thermo Fisher Scientific). A library concentration of at least 3–5 ng/µl was required to achieve efficient bead-based library normalization. Normalized libraries were pooled together (maximum of eight libraries per pool incubated at 96 °C for 2 min). Two different dilutions were performed to obtain a final concentration of 1.5 pM. Sequencing was performed on NextSeq® 500 platform using High Output reagents, 8 samples per flowcell and 300 cycles of sequencing in paired-end (Illumina). Furthermore, PhiX control at 1% (Illumina) was used as a sequencing control.

#### QIAseq® targeted DNA panel

The QIAseq® Tumor Mutation Burden Panel targets relevant mutations in 486 genes related to tumor and covers a total 1.3 Mb of DNA with single-primer extension technology (Qiagen). Libraries were prepared using 40 ng of gDNA following the manufacturer’s instructions. The generated libraries were run on 2100 Bioanalyzer Instrument (Agilent) using the High Sensitivity DNA Kit (Agilent). The sequencing run was performed on NextSeq® 500 using high output reagents and 300 cycles of sequencing in paired-end (Illumina) and custom primers for read1. Each high output flowcell contained 8 samples, while the PhiX control at 1% (Illumina) was used as a sequencing control.

#### FoundationOne CDx

F1CDx (Foundation Medicine, Cambridge, MA, USA) was used as the reference standard method in our EQA scheme, based on the results of the KEYNOTE 158 trial [16]. The assay covers the exonic regions from 324 cancer-related genes and selected introns from 51 genes commonly rearranged in cancer for a total coverage of 0.8 Mb of DNA. Two 10-µm slides from each of the nine cell lines were sent to Roche FMI (Penzberg, Germany) for the analysis.

## Results

### Validation of control material

The first objective of the EQA pilot scheme was the identification and validation of adequate control material for TMB estimation. In this respect, several different commercial reference material manufacturers were invited through a tender. Special requirements for the supply of samples were specified, including the potential yield of DNA, variability of TMB values, and availability of cell lines as FFPE blocks. Seracare (LGC Clinical Diagnostics, Inc.) was awarded the supply contract, and the IQNPath panel of experts selected from the list provided the following nine cell lines: *NCI-H2009*, *NCI-H2126*, *NCI-H2171*, *NCI-H1437*, *NCIH23*, *NCIH322*, *SKMEL2*, *C33A*, and *IGROV1*.

**Fig. 1:**
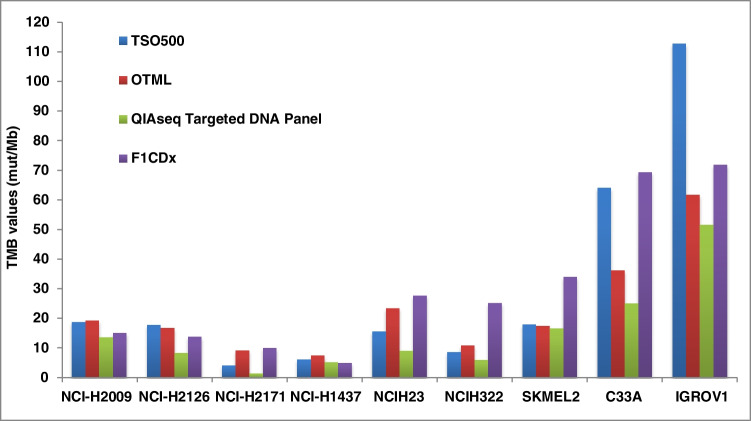
Results of the internal validation phase on 9 cell lines. The TMB values were calculated using three different commercial NGS panels (OTML, TSO500, and QIAseq Targeted DNA Panel) and compared with the reference F1CDx assay

We performed a validation phase on the selected cell lines using three different commercial NGS panels (see “Materials and Methods”; report files are uploaded at link: 10.5281/zenodo.6563860). Two independent gDNA extractions with the GeneRead™ DNA FFPE Kit (Qiagen) obtained comparable results in terms of gDNA yield (data not shown). Similarly, two independent NGS runs using the OTML, TSO500, and QIAseq Targeted DNA Panel were conducted to ensure the consistency of the results. The FFPE cell lines were also tested with the F1CDx assay (reported in figures and tables as FMI). The results are summarized in Fig. 1.

All the samples returned valid TMB results with the four NGS panels, and between the two parallel runs, the standard deviation between the same panel was very low (0.01–5.07; 0–6.98; 0–0.54; 0–7.13 for TSO500, OTML, QIAseq, and F1CDx, respectively). However, there were significant differences between the TMB scores obtained from the four NGS assays (Fig. 1 and Supplementary Table 1). In particular, the concordance of the TMB values was low among the TSO500, OTML, and QIAseq assays and between these panels and the reference F1CDx test. Although the cutoff of the different tests is likely to be different, the important differences found could result in a misclassification of the samples in high vs low TMB.

Following this validation phase, five cell lines, *NCI-H2171* (*TMB1*), *NCI-H1437* (*TMB2*), *NCI-H2126* (*TMB3*), *NCI-H2009* (*TMB4*), and *C33A* (*TMB5*), covering a TMB range “low to high,” were chosen as the EQA materials. Before shipment of the samples to the participating centers, two additional runs were performed on these five samples from two new independent gDNA extractions (Supplementary Fig. 1). These results confirmed the data of the initial validation. Finally, thirty laboratories were selected to participate in the pilot EQA through a survey, the results of which have been recently published. These laboratories were selected on the basis of several criteria including their expertise and experience in NGS technologies and the method they used to assess TMB [29].

### TMB testing methods of participating laboratories

Twenty-nine laboratories confirmed their participation in the EQA scheme and received the samples, specifically from twelve different countries registered with AIOM (*N* = 3), ESP (*N* = 4), EMQN (*N* = 14), GenQA (*N* = 6), and Gen&Tiss (*N* = 2). Twenty-three (79.3%) laboratories submitted results. One laboratory submitted results using two different panels (Lab6 and Lab6bis). Out of 29 participating laboratories, two (6.9%) withdrew from the scheme because of the absence of “normal” (non-tumor tissue) matched samples, and four (13.8%) did not submit results without providing any explanation. All participating laboratories provided details of the DNA extraction and NGS methodology that they used to assess TMB. Eleven different commercially available kits for DNA extraction from FFPE tissue were used for DNA extraction (Table 1). The GeneRead FFPE DNA kit (Qiagen) was the most popular kit followed by the QIAmp DNA FFPE kit (Qiagen). Notably, no participating laboratory reported a critical issue in this preanalytical phase, confirming the results obtained in the validation phase of the EQA and suggesting that sufficient good-quality DNA was present in the samples.

**Table 1:** Commercially available kits used for DNA extraction

Extraction kit	N° of laboratories
GeneRead DNA FFPE	5
QIAamp DNA FFPE	4
Maxwell 16 FFPE Plus LEV DNA	3
Allprep FFPE RNA/DNA	2
Maxwell RSC DNA FFPE	2
QIAsymphony DSP DNA	1
MagCore Genomic DNA	1
Maxwell 16 CSC FFPE DNA	1
MagMax FFPE DNA/RNA Ultra	1
Arcturus PicoPure DNA	1
Cobas DNA Sample Prep	1

A wide array of NGS panels and methods for library preparation were employed by the participating laboratories (Table 2). The majority of participating laboratories used a targeted sequencing approach with multigene testing panels. Only two laboratories performed WES and two assessed TMB by clinical exome. The OTML was the most widely used commercially available panel, although five laboratories used custom panels. The characteristics of the NGS methods are summarized in Supplementary Table 2.

**Table 2:** Next Generation Sequencing panels used for TMB test by participating laboratories

Panel	N° of laboratories
Oncomine Tumor Mutation Load	6
Custom panel	5
TruSight Oncology 500	4
Oncomine Comprehensive Assay Plus	3
SureSelect XT-HS	1
Twist Human Exome plus RefSeq	1
IDT xGen Pan-Cancer Panel v2.4	1
Oncomine Comprehensive Cancer	1
Nimblegen SeqCap EZ MedExome	1

### Assessment of the performance of participating laboratories

**Fig. 2:**
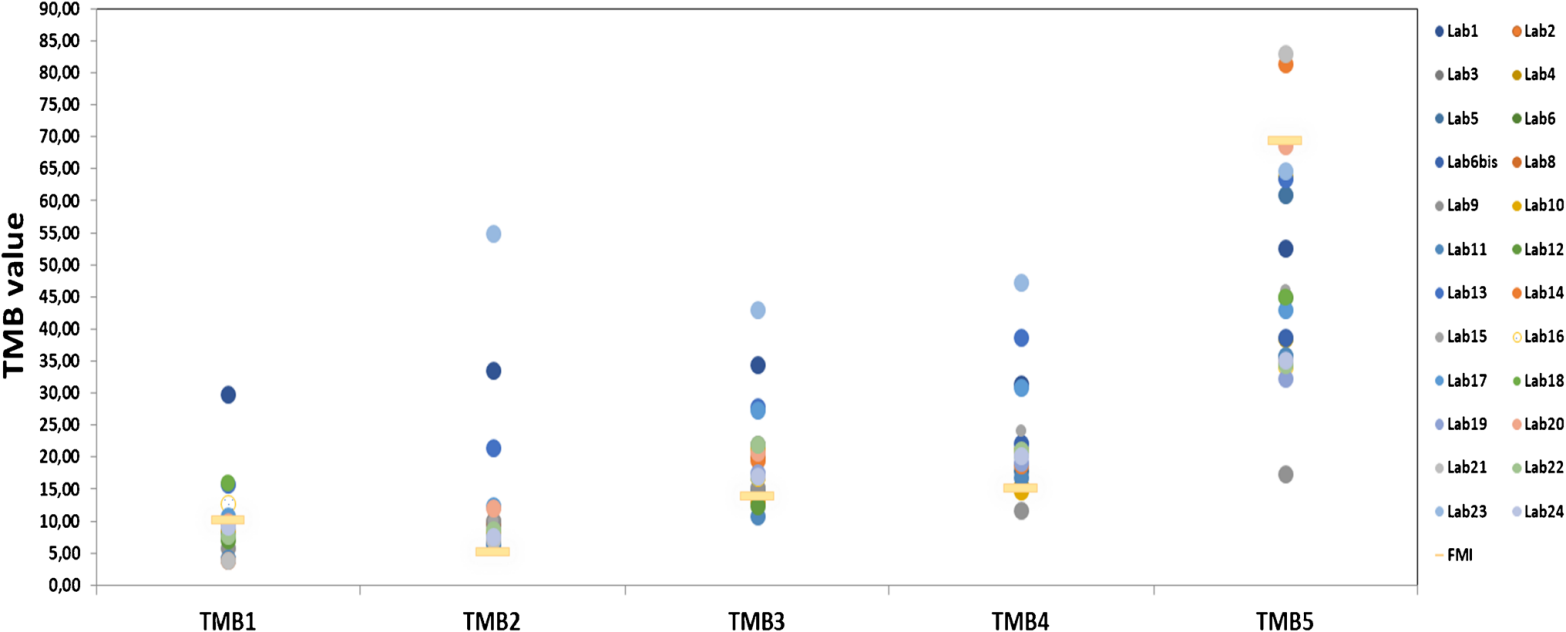
Summary of results for TMB test submitted by different laboratories. The five selected samples were tested by participating laboratories using different methods. The circles represent the TMB value reported by each participating center, while the horizontal bar indicates the TMB value obtained with the reference F1CDx assay

A general overview of the results submitted and compared with the TMB values assessed by the F1CDx test is shown in Table 3 and Fig. 2. As expected, the use of different technologies for TMB testing led to significant variability in the reported TMB values. The variability was higher for sample TMB5, which had the highest TMB value according to the F1CDx test, when compared to the other testing methods. All the laboratories reported higher TMB values for sample TMB2 as compared with the F1CDx result, which was confirmed by a repeated analysis with the F1CDx panel. Importantly, excluding outliers, good reproducibility of the TMB score was shown by laboratories using the same panel. In this respect, the aim of the pilot was achieved, and the commercial material used appears suitable as a reference for TMB evaluation using a variety of different testing methods. The majority of laboratories did not indicate a TMB cut-off value, and when a value was reported, it was not clear if the cut-off value provided was effectively validated. Therefore, it was not possible to provide any feedback about the clinical interpretation of the TMB value. Due to the challenging nature of this biomarker, no scoring of the results from the participating laboratories was applied. The assessment process has been conducted by benchmarking the performance of each laboratory in two different ways: comparing the reported results against the reference F1CDx test and comparing the reported results against other centers using the same method.

**Table 3:** Summary of results for TMB test submitted by different laboratories

Lab N°	TMB1	TMB2	TMB3	TMB4	TMB5
Lab1	29.9	33.51	34.54	31.44	52.58
Lab2	9.29	7.61	16.96	21.1	35.34
Lab3	8.57	9.54	21.95	20.83	35.65
Lab4	7.8	8.5	13	15.6	38.5
Lab5	4	7	15	18	61
Lab6	8.59	10.74	21.01	22.06	34.6
Lab6bis	8.04	6.92	17.13	22.16	38.66
Lab8	NA	10	20	NA	35
Lab9	5.82	10.19	15.29	11.65	17.48
Lab10	8.62	8.11	21.3	14.7	63.9
Lab11	4.25	6.36	10.81	16.92	35.94
Lab12	7	NA	12.5	21	45
Lab13	15.77	21.51	27.96	38.71	63.44
Lab14	3.9	7.8	19.6	18.8	81.5
Lab15	12.56	10.03	20.91	24.22	45.99
Lab16	12.71	7.6	16.85	20.99	34.16
Lab17	10.74	12.36	27.36	30.94	42.98
Lab18	15.99	7.59	16.81	20.22	44.98
Lab19	9.2	7.53	17.6	19.23	32.32
Lab20	10	12.14	20.71	20.71	68.57
Lab21	3.9	7.1	NA	20.4	83
Lab22	7.78	8.61	22.1	21.16	34.4
Lab23	NA	54.9	42.93	47.21	64.66
Lab24	9.27	7.58	16.97	20.23	35.12
FMI	10.09	5.04	13.87	15.13	69.35

*NA* not assessed

The results submitted by laboratories using the OTML showed good inter-laboratory reproducibility, although some variability was observed; such variability was higher in the case of TMB5 compared with other samples (Supplementary Fig. 2). To better understand the reason for this variability, we analyzed the results taking into account the bioinformatics pipeline used by the laboratories to call TMB. In particular, four laboratories (Lab 2, Lab 6bis, Lab 19, and Lab 24) included only non-synonymous mutations in the assessment of TMB, whereas three centers (Laboratories 16, 17, and 18) used both non-synonymous and synonymous variants. Dividing the laboratories into two groups taking into consideration the different pipelines used to call TMB, we observed a lower inter-laboratory variability in the group of laboratories that included only non-synonymous variants. In addition, the values reported by these centers were closer to values of F1CDx as compared to the values of the laboratories that used both synonymous and non-synonymous alterations for TMB estimation (Table 4). The recommended TMB evaluation algorithm for the OTML panel includes only non-synonymous variants.

**Table 4:** TMB scores reported with OTML panel using the different bioinformatics pipeline

	TMB1	TMB2	TMB3	TMB4	TMB5
Non-synonymous only					
Lab2	9.29	7.61	16.96	21.1	35.34
Lab6bis	8.04	6.92	17.13	22.16	38.66
Lab19	9.2	7.53	17.6	19.23	32.32
Lab24	9.27	7.58	16.97	20.23	35.12
Std	0.53	0.28	0.26	1.08	2.25
Non-synonymous and synonymous					
Lab16	12.71	7.6	16.85	20.99	34.16
Lab17	10.74	12.36	27.36	30.94	42.98
Lab18	15.99	7.59	16.81	20.22	44.98
Std	2.16	2.24	4.96	4.88	4.70
Validation phase	9.24	7.54	16.81	19.31	36.23
FMI	10.09	5.04	13.87	15.13	69.35

*Std* standard deviation

Regarding the three laboratories that used the Oncomine Comprehensive Assay Plus (OCA Plus), an almost perfect reproducibility of the results for all the samples was observed. In fact, the submitted results showed a very low standard deviation ranging between 0.38 and 0.87 (data not shown). Nevertheless, the TMB value obtained with OCA Plus in 3/5 samples was overestimated as compared to FMI values (Supplementary Fig. 3). On the other hand, for laboratories that used the TSO500 panel, excluding a clear outlier (Lab 1), they presented a good inter-laboratory reproducibility. However, all the laboratories (apart from Lab 1) using the TSO500 underestimated the TMB value of TMB1, and they also showed a greater variability for TMB5 (Supplementary Fig. 4). Of the two labs that used WES, Lab 23 overestimated TMB for 3 out of four samples as compared to F1CDx, whereas the results submitted by Lab 11 were in line with the reference score (Supplementary Fig. 5). The laboratories that performed “Clinical exome” testing showed results very close to the F1CDx values even if they overestimated TMB2 and TMB4 samples (Supplementary Fig. 6). Lastly, for laboratories which used unique custom panels, they submitted results similar to the F1CDx score. For this group of laboratories, it is important to note a higher variability for TMB5 and the overestimation for TMB2 (Supplementary Fig. 7).

## Discussion

There is an ongoing debate regarding the role of TMB as a predictive biomarker for immunotherapy. The inconsistency of results of clinical trials raises several questions about the ability of this biomarker to identify patients most sensitive to immunotherapy. On the other hand, the studies that have evaluated different biomarkers for immunotherapy include TMB among those most related to its efficacy [30, 31]. The response to immunotherapy depends on the characteristics of the tumor cells, including TMB, but also on those of the tumor microenvironment. Therefore, TMB is probably not sufficient to predict response to immunotherapy as a single biomarker, but it needs to be combined with the features of the tumor microenvironment [32]. In agreement with this hypothesis, high TMB levels correlated with response to immunotherapy only in cancer types where CD8 T-cell levels positively correlated with neoantigen load, including melanoma, lung, and bladder cancers [33].

Although TMB has only recently been approved by the FDA as an agnostic marker for pembrolizumab, several laboratories around the world have been offering this test for a significant period of time [29]. This phenomenon is probably related to the use in many academic centers of large panels for the identification of actionable mutations that allow enrollment of patients in clinical trials [34]. Large sequencing panels also provide information on complex biomarkers such as TMB, which are therefore described in the clinical reports.

In this assessment, we found high inter-laboratory variability in TMB testing as compared with the F1CDx assay. We chose this test as a reference since the majority of clinical trials that assessed the TMB as a biomarker used the F1CDx assay for TMB testing, including the KEYNOTE-158 study that led to the approval of TMB as an agnostic biomarker for pembrolizumab [16]. Other studies reported a similar inter-laboratory variability using a variety of either commercially available or academic-targeted sequencing panels [35–37].

Importantly, the inter-laboratory variability among centers that used the same panel for TMB assessment was limited in our study, thus underlying a good quality of the laboratories’ workflow of analysis. However, some outliers were found, for example, among laboratories using WES or TSO500 (Supplementary Figs. 4 and 5). While WES is still far from standardization for clinical use at least for TMB testing, TSO500 is an assay for comprehensive genomic profiling with a well-defined workflow of analyses. However, we must acknowledge that the TSO500 was available for a short time when this EQA scheme was launched, and this might reflect the low level of expertise in some laboratories. An important source of variability is represented by bioinformatics analyses. In this regard, the laboratories that included both synonymous and non-synonymous variants in the TMB calculation with the OTML panel reported greater variability than those that included only the non-synonymous variants. This result may have been predicted based on previous data also produced by our laboratory [38]. Furthermore, the manufacturer suggests the calculation of the TMB with only the non-synonymous variants.

Notably, a high variability in TMB scores was found in samples TMB2 and TMB5. In particular, for TMB2, all the laboratories overestimated the TMB value, as compared with F1CDx. An even higher variability was found for TMB5, although all the centers calculated a TMB value higher than the F1CDx cut-off of 10 mut/Mb. Several factors are likely to affect the TMB calculation, including exons coverage, genes analyzed and if they are covered only in hotspot or full coding regions, sequencing technologies, and bioinformatics pipelines. The identification of a cut-off to separate high-TMB patients, who are likely sensitive to immunotherapy, from low-TMB immunotherapy-resistant patients still represents an unresolved issue. The KEYNOTE-158 trial used a cut-off of 10 Mut/Mb to separate high-TMB from low-TMB patients [16]. Evidence suggests that the optimal cut-off correlating with the activity of immunotherapy may be different among the various cancer types [25, 26]. In addition, the cut-off may be different between panels covering different numbers and types of genes and using different bioinformatics analyses [26]. In this respect, a cut-off should be likely identified for each panel and validated by the testing laboratories. Indeed, the majority of laboratories that participated in this pilot EQA scheme did not indicate any cut-off and did not make any clinical interpretation of the results. However, describing TMB values in a report without identifying a cut-off for the test used may lead the clinician to misclassify patients and assign an inappropriate treatment based on the F1CDx cut-off. Taken together, these findings confirm the need to continue the process of harmonization and standardization of TMB testing but also highlight the necessity of additional research to fill the gaps still existing on the biological phenomena behind TMB and their implications in sensitivity to immunotherapy. In this respect, several studies have been recently published demonstrating significant differences among commercial panels in TMB estimation of the same samples [28, 35, 37]. Adjusted cut-off values of 7.847 mutation/Mb for TSO500 and 8.380 mutations/Mb for OTML have been also proposed to gain a sensitivity > 88% [28]. However, these cutoffs are not used by most laboratories and still need clinical validation.

Our study also confirms the importance of EQA for the assessment of test quality. In fact, some outliers were identified among laboratories using the same method, suggesting the need for continuous external quality control to ensure high standards for biomarker testing. In this respect, the different laboratories participating in this initiative had the opportunity to test the same control samples provided to other centers and to compare their performance in a continuous process of improving the quality of their tests. EQA programs also play a central role in the context of the new IVDR regulation [39].

Finally, this program provides relevant information for the future development of EQA for complex biomarkers. Most EQA programs have been focused up until now on specific genomic alterations that are relatively simple to test and interpret. However, more complex biomarkers are entering into clinical practice, including TMB, microsatellite instability (MSI), and homologous repair deficiency (HRD). The development of EQA programs for such complex biomarkers presents considerable difficulties for the selection of materials, validation procedures, and the interpretation of the results. In this context, we have developed a methodology which involves the careful selection of a material provider, a preliminary analysis of a large panel of samples, and the selection and final validation of a smaller set of samples with specific characteristics resembling the spectrum of values observed in clinical practice.

In conclusion, this pilot EQA scheme suggests that it is feasible to run such an EQA program for TMB assessment. However, the results of our pilot highlight the numerous challenges for the determination of this biomarker, which require both a greater standardization of the test and further research on the biological mechanisms underlying the mutational load of a tumor and its correlation with the activity of ICIs.

## Supplementary Information

Below is the link to the electronic supplementary material.

Supplementary file1 (PDF 260 kb)

Supplementary file2 (PDF 113 kb)
