# Correction to: External quality assessment (EQA) for tumor mutational burden: results of an international IQN path feasibility pilot scheme

**DOI:** 10.1007/s00428-022-03474-6

**Published:** 2022-12-09

**Authors:** Riziero Esposito Abate, Melanie H. Cheetham, Jennifer A. Fairley, Raffaella Pasquale, Alessandra Sacco, Wolstenholme Nicola, Zandra C. Deans, Simon J. Patton, Nicola Normanno

**Affiliations:** 1 Cell Biology and Biotherapy Unit, Istituto Nazionale Tumori “Fondazione G. Pascale”-IRCCS, Naples, Italy; 2 European Molecular Genetics Quality Network (EMQN), Unit 4, Enterprise House, Pencroft Way, Manchester Science Park, Manchester, M15 6SE UK; 3 GenQA, Department of Laboratory Medicine, Royal Infirmary of Edinburgh, Little France Crescent, Edinburgh, EH16 4SA UK

**Correction to: Virchows Archiv**

10.1007/s00428-022-03444-y

In the original published version of the above article contained incorrect Table legend. The Table 2 with correct legend is as follows:

**Table 2:** Next Generation Sequencing panels used for TMB test by participating laboratories

Panel	N° of laboratories
Oncomine Tumor Mutation Load	6
Custom panel	5
TruSight Oncology 500	4
Oncomine Comprehensive Assay Plus	3
SureSelect XT-HS	1
Twist Human Exome plus RefSeq	1
IDT xGen Pan-Cancer Panel v2.4	1
Oncomine Comprehensive Cancer	1
Nimblegen SeqCap EZ MedExome	1

The original article has been corrected.

