# Supplementary material for: External quality assessment (EQA) for tumor mutational burden: results of an international IQN path feasibility pilot scheme
Source: Virchows Arch. 2022 Nov 10;482(2):347–55. doi: 10.1007/s00428-022-03444-y (PMC9931778; doi:10.1007/s00428-022-03444-y)

Supplementary Figure 1 -Results of the internal validation phase before ship to participating laboratories

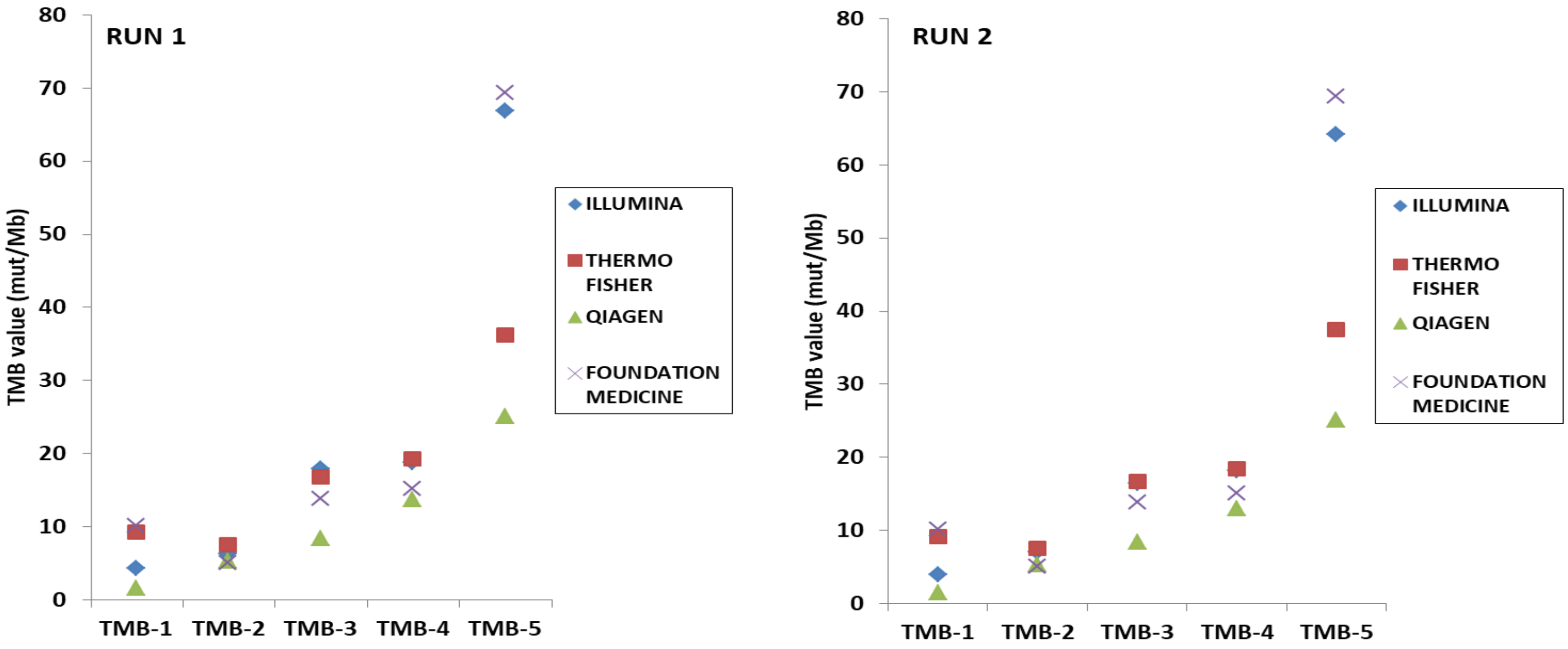

Supplementary Figure 2 - Summary of TMB results for OTML test submitted by different laboratories

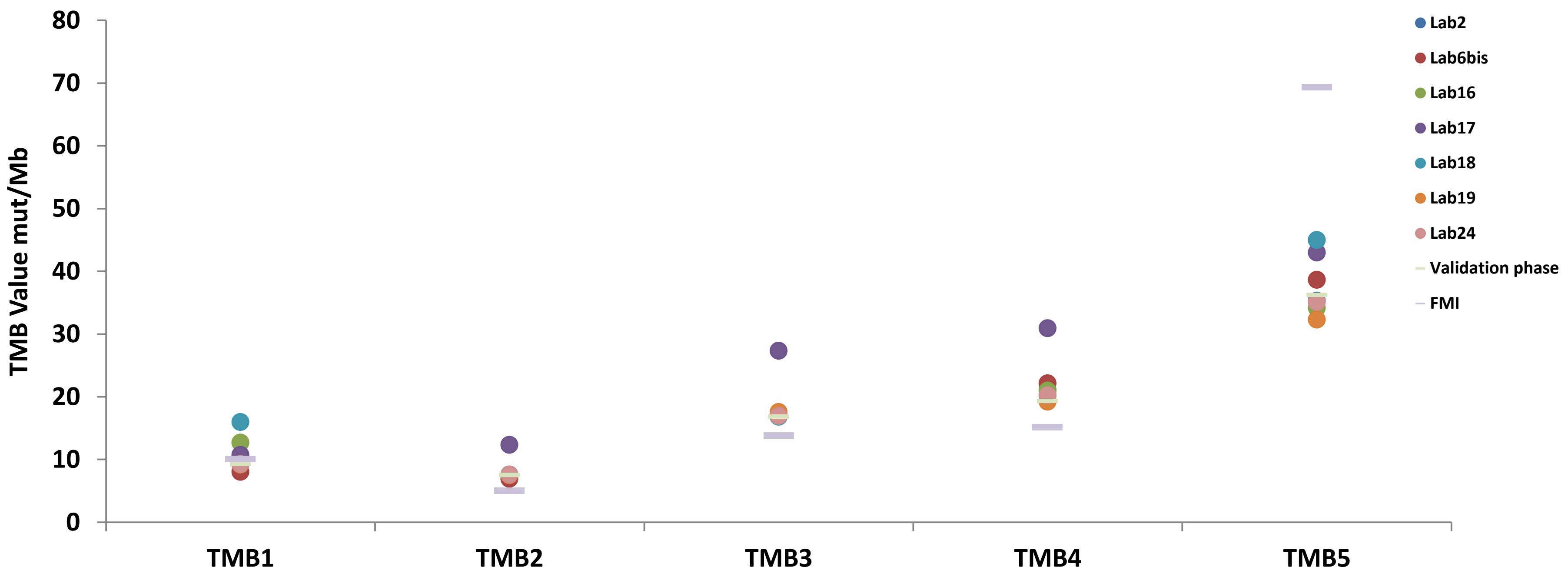

Supplementary Figure 3 - Summary of TMB results for OCA Plus test submitted by different laboratories

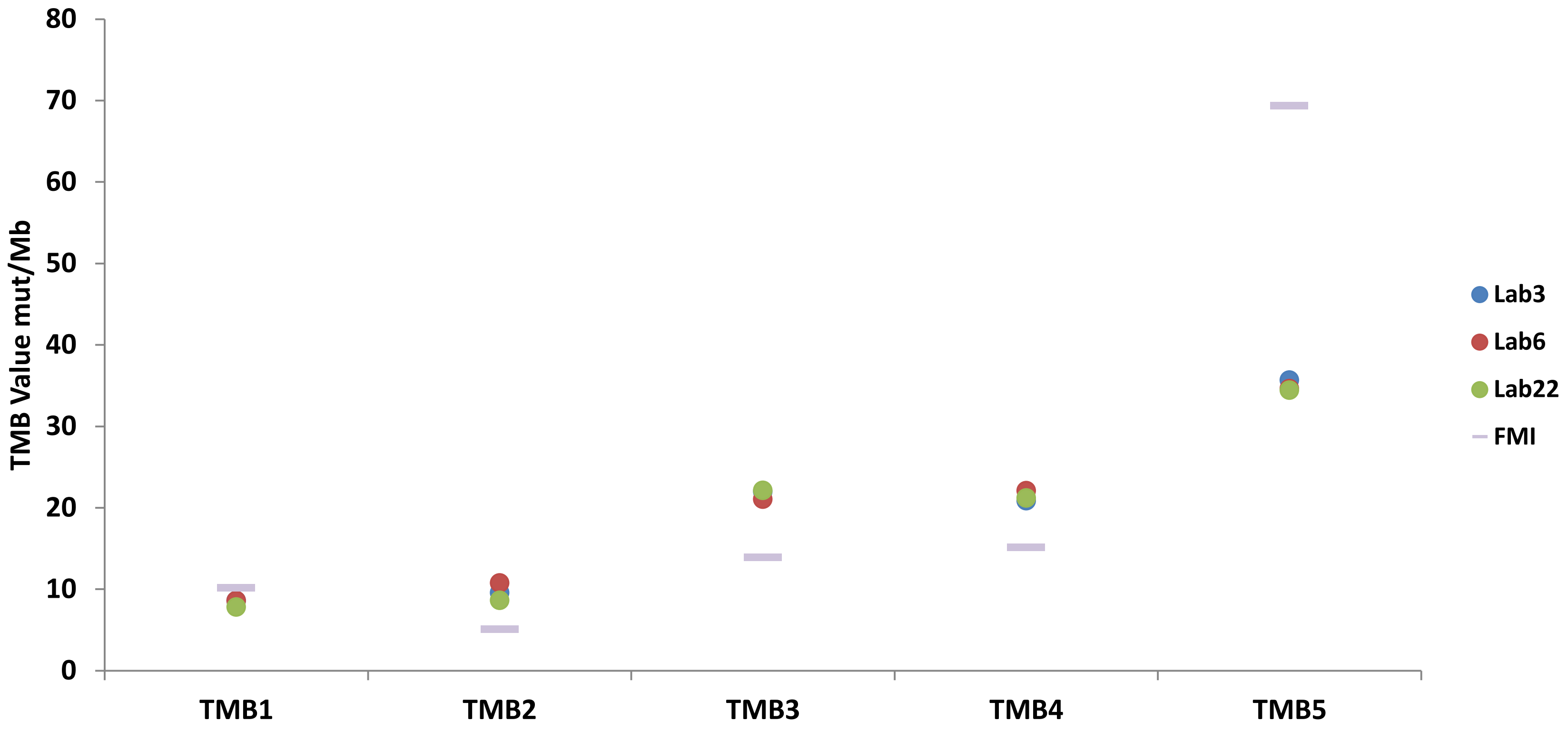

Supplementary Figure 4 - Summary of TMB results for TSO500 assay submitted by different laboratories

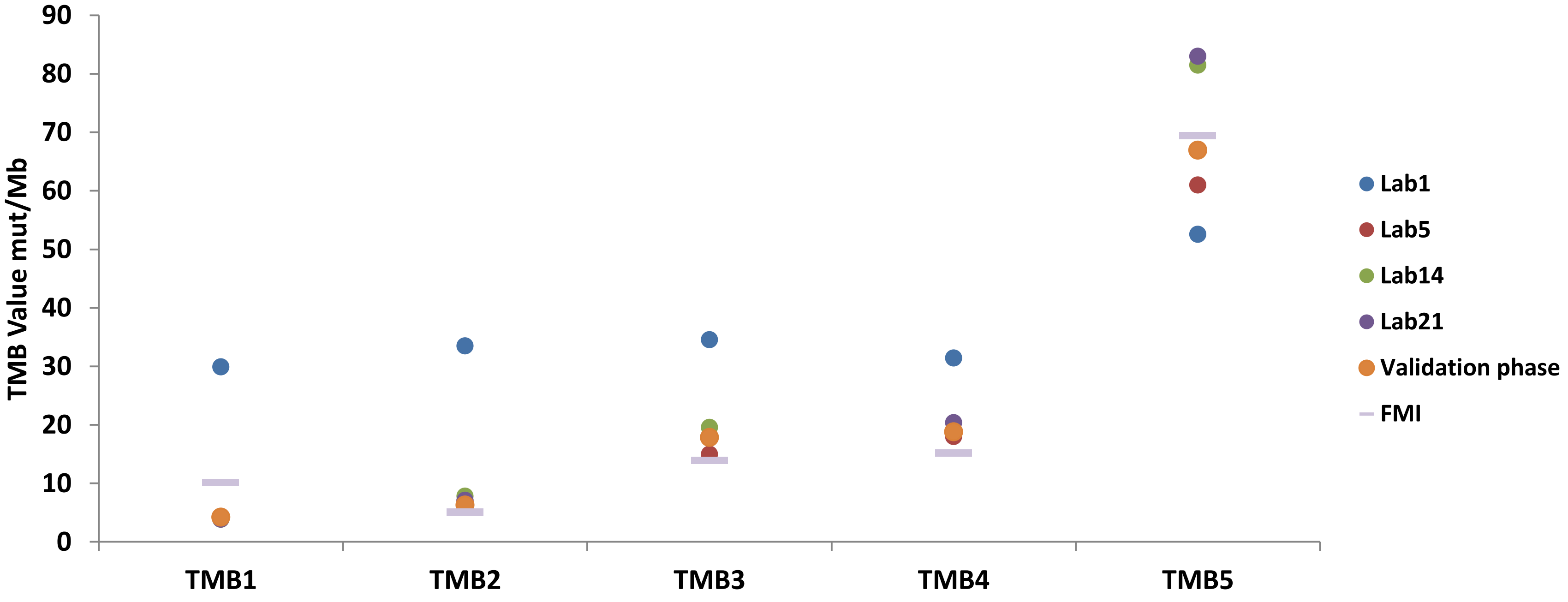

Supplementary Figure 5 - Summary of TMB results for WES approach submitted by different laboratories

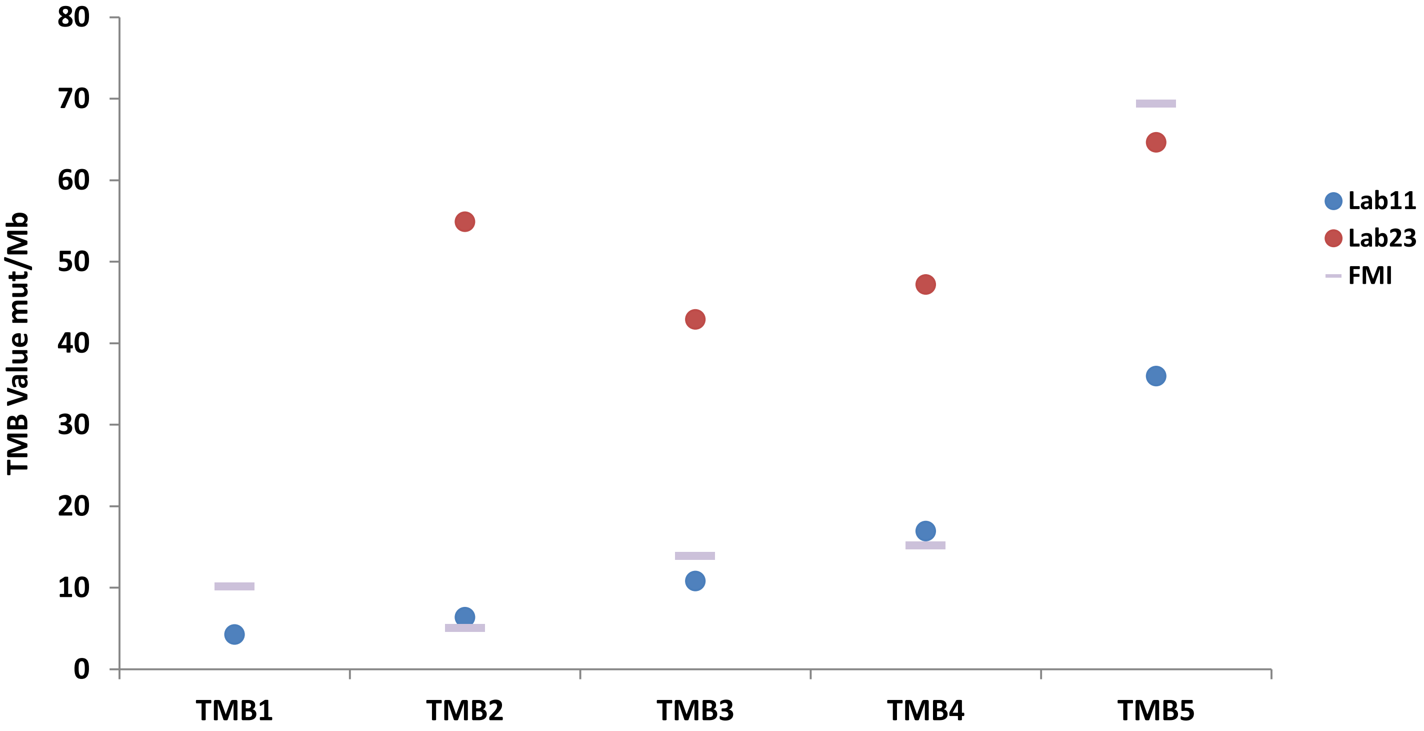

Supplementary Figure 6 - Summary of TMB results for clinical exome by different laboratories

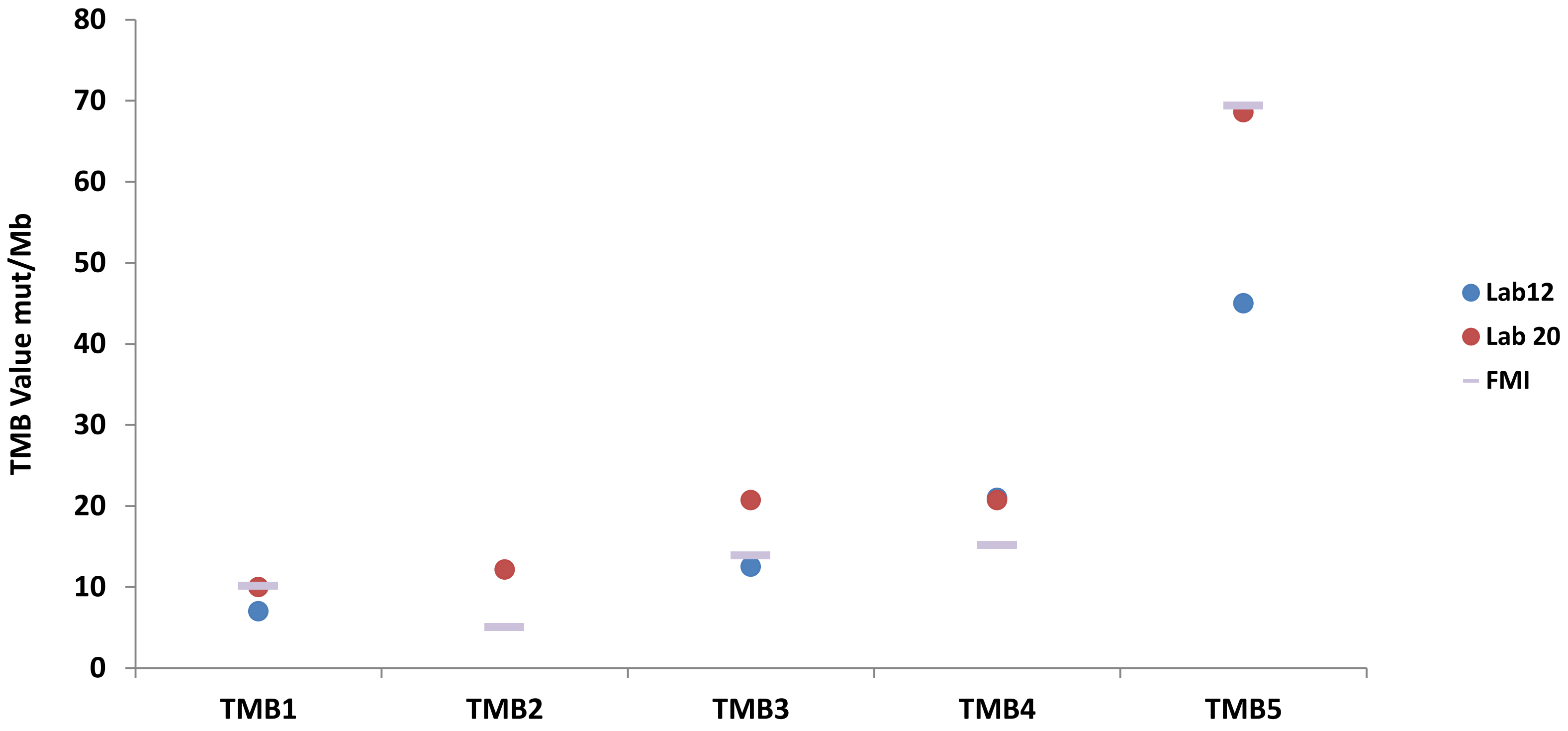

Supplementary Figure 7- Summary of results for TMB test with single method submitted by different laboratories

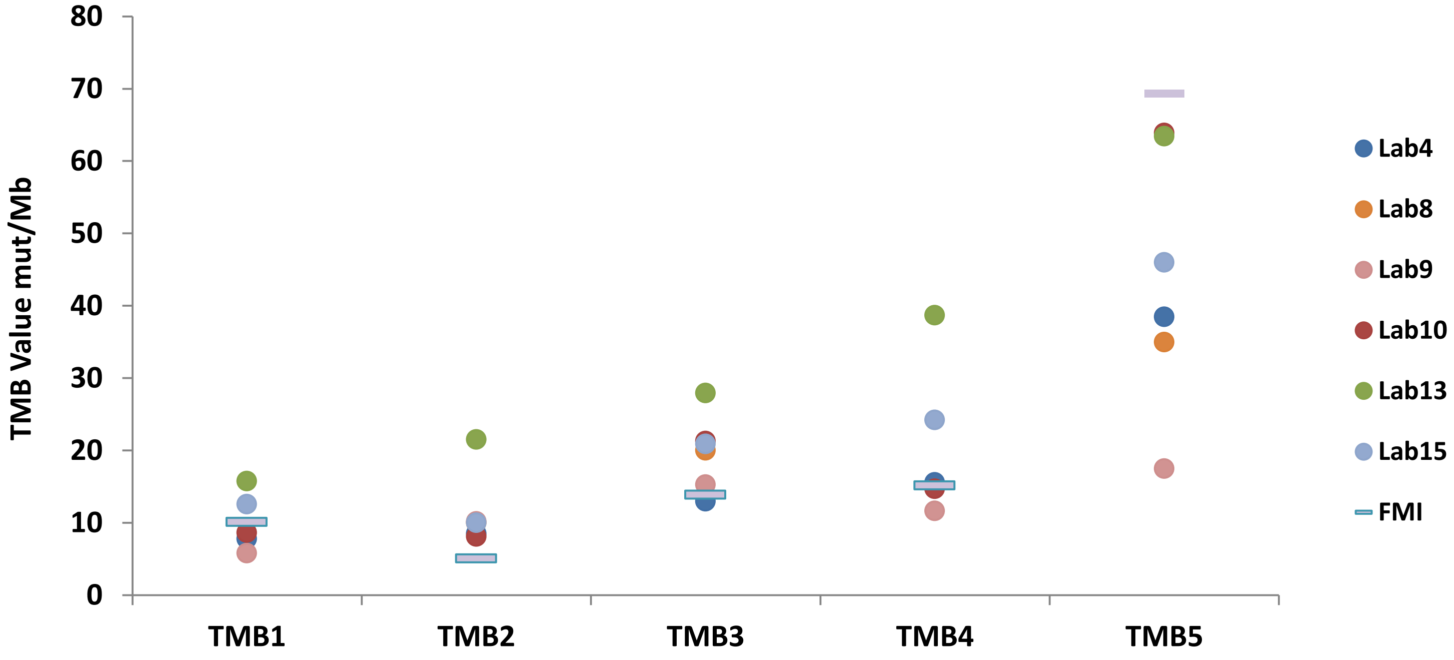

Supplement: Supplementary file 1 — Supplementary file1 (PDF 260 kb) [file 428_2022_3444_MOESM1_ESM.pdf]
