# Supplementary material for: External quality assessment (EQA) for tumor mutational burden: results of an international IQN path feasibility pilot scheme
Source: Virchows Arch. 2022 Nov 10;482(2):347–55. doi: 10.1007/s00428-022-03444-y (PMC9931778; doi:10.1007/s00428-022-03444-y)
Supplement: Supplementary file 2 — Supplementary file2 (PDF 113 kb) [file 428_2022_3444_MOESM2_ESM.pdf]

Supplementary Table 1 – Results of the internal validation phase from 2X parallel runs for 4 different NGS panels for 9 tested cell lines.

Cell line	Illumina		ThermoFisher		Qiagen		Foundation Medicine	
	**RUN1**	**RUN2**	**RUN1**	**RUN2**	**RUN1**	**RUN2**	**RUN1**	**RUN2**
**NCI-H2009**	18.801	18.147	19.31	18.41	13.69	12.92	15.13	15.13
**NCI-H2126**	17.864	16.479	16.81	16.74	8.38	8.38	13.87	13.87
**NCI-H2171**	4.248	3.92	9.24	9.19	1.52	1.52	10.09	10.09
**NCI-H1437**	6.282	7.058	7.54	7.56	5.33	5.33	5.04	5.04
**NCI-H23**	15.667	13.298	23.5	24.26	9.11	9.88	27.74	27.74
**NCI-H322**	8.651	8.632	10.9	10.9	6.09	5.32	25.22	25.22
**SKMEL2**	18.003	25.174	17.56	17.53	16.71	16.72	34.04	44.13
**C33A**	66.942	64.147	36.23	37.49	25.08	25.08	69.35	69.35
**IGROV1**	112.715	113.429	71.6	61.73	51.66	51.66	71.87	76.91

Supplementary Table 2 – Description of the participating NGS methods at the pilot EQA scheme

Company	Panel Name	Type of NGS approach	Type of variants used to infer TMB	Number of gene
**IDT**	Custom panel	Gene targated	Non synonimous mutations, Indels	423
**Twist Bioscience**	Custom panel	Gene targated	Non-synonymous mutations, Splice variants, Indels	350
**Twist Bioscience**	Custom panel	Gene targated	Synonymous and non-synonymous mutations, Indels	645
**Agilent**	Custom panel	Gene targated	Non synonimous mutations, Indels	431
**Illumina**	TruSight Oncology 500	Gene targated	Non synonimous mutations, Indels	523
**Thermo Fisher Scientific**	Oncomine Tumor Mutation Load	Gene targated	Non synonimous and synonimous mutations, Indels	409
**Thermo Fisher Scientific**	Oncomine Tumor Mutation Load	Gene targated	Non synonimous mutations, Indels	409
**Thermo Fisher Scientific**	Oncomine Comprehensive Assay Plus	Gene targated	Non synonimous mutations, Indels	514
**IDT**	IDT xGen Pan-Cancer Panel v2.4	Clinical exome	Non synonimous mutations	523
**Agilent**	Custom Panel	Clinical exome	Non synonimous mutations, Indels	431
**Twist Bioscience**	Twist human Exome plus	Whole exome sequencing	Non-synonymous mutations, Splice variants, Indels	22000
**Roche**	Nimblegen (Roche) SeqCap EZ MedExome	Whole exome sequencing	Synonymous and non-synonymous mutations, Indels	22334
